# 2 Cases of Bullosis Diabeticorum following Long-Distance Journeys by Road: A Report of 2 Cases

**DOI:** 10.1155/2012/367218

**Published:** 2012-10-16

**Authors:** Fatima Bello, O. Modupe Samaila, Yakubu Lawal, U. Kufre Nkoro

**Affiliations:** ^1^Endocrine Unit, Department of Medicine, Ahmadu Bello University Teaching Hospital, 810001 Zaria, Nigeria; ^2^Department of Pathology, Ahmadu Bello University Teaching Hospital, 810001 Zaria, Nigeria

## Abstract

*Background*. Bullosis diabeticorum is a distinct, spontaneous, noninflammatory, and blistering condition of acral skin that is unique to diabetics. It is rare. Exact aetiopathogenesis is not known, but many attributed peripheral neuropathy as a potent risk factor, others hypothesized the role of trauma, UV light, and nephropathy. *Aim*. To present cases of bullosis diabeticorum following long-distance journeys by road. *Methods*. History and physical examinations were done on 2 diabetics who presented with bilateral feet bullae following a long journey. Biopsy of a circumferential area of the bullae including adjoining apparently normal skin was done. *Results*. Features of peripheral neuropathy were noted. One developed digital gangrene without features of peripheral vascular disease. Culture of aspirate from a bullae yielded *Staphylococcus aureus*. Tissue biopsy showed hyperkeratotic focally acanthotic pigmented epidermis with subcorneal separation of the granular layer of the epidermis by aggregates of viable and nonviable polymorphs and lymphocytes. There is mild acantholysis of the epidermis, and a fibrocollagenous dermis which is moderately infiltrated by lymphocytes. *Conclusion*. Long journeys by road is a strong factor in the aetiopathogenesis of bullosis diabeticorum on a background of peripheral neuropathy. Diabetics especially those with peripheral neuropathy should be cautious while traveling long journeys by road.

## 1. Background

Bullous disease of diabetes (bullosis diabeticorum) is a distinct, spontaneous, noninflammatory, and blistering condition of acral skin that is unique to patients with diabetes mellitus [1].

Kramer first reported bullous-like lesions in diabetic patients in 1930 [2]; Rocca and Pereyra first characterized this as a phlyctenar (appearing like a burn-induced blister) in 1963 [3]. Cantwell and Martz are credited with naming the condition bullosis diabeticorum in 1967 [4]. It is also termed bullous disease of diabetes and diabetic bullae.

Bullous disease of diabetes tends to arise in long-standing diabetes or in conjunction with multiple complications. Prominent acral accentuation of bullous disease of diabetes lesions suggests a susceptibility to trauma-induced changes, but the definitive explanation awaits elucidation [1].

In the United States, bullous disease of diabetes has been reported to occur in approximately 0.5% of diabetic patients. Male patients have twice the risk as female patients [1].

While lesions typically heal spontaneously within 2–6 weeks, they often recur in the same or different locations. Secondary infections may also develop; these are characterized by cloudy blister fluid and require a culture [1].

The clinician should consider direct immunofluorescence studies to exclude histologically similar entities (e.g., noninflammatory bullous pemphigoid, epidermolysis bullosa acquisita, porphyria cutanea tarda, and other bullous porphyrias).

Specific treatment is unwarranted unless secondary infections (i.e., staphylococcal) occur, thereby warranting antibiotic therapy. However, aspiration of fluid from lesions using a small-bore needle might help prevent accidental rupture [1].

**Aim**

To present and describe 2 cases of diabetic bullae following long journeys by road.

**Objectives**

1.   To describe 2 cases of diabetic bullae following long journeys in tightlypacked commercial vehicles.
2.   To show the presence of peripheral neuropathy in these cases as have been described by previous case reports.
3.   To show the presence of digital gangrene in one of the cases after the development of the bullae with historical background and clinical evidence of neuropathy.
4.   To suggest the role of pressure, vibration/Raynaud's phenomenon, and mild to moderate heat in a background of peripheral neuropathy in the pathogenesis of diabetic bullae.

## 2. Case 1

A. M. is a 59-year-old man who developed sudden onset of progressively enlarging swelling on both feet immediately following a 10-hour journey in a tightlypacked bus. He initially was unable to walk out of the bus due to sudden weakness of both lower limbs with marked numbness but no pain and was later supported out of the bus. Initially, swelling was painless but later became mildly painful as the swelling increased in size. He experienced numbness and pins and needles sensation in gloves and stocking distribution for the previous one year. He has no history of intermittent claudication and no frothiness of urine but was said to have experienced deteriorating vision for the previous one year. A known type 2 diabetic diagnosed 11 years ago on account of osmotic symptoms was regular on antidiabetic (metformin,) and subsequently controlled on subcutaneous mixtard insulin. He was also diagnosed hypertensive one year prior to presentation and was placed on Nifedipine XL 30 mg daily Lisinopril 10 mg daily but not regular on clinic visit or medication. He has never smoked a cigarette or ingested alcohol.

Anthropometric examination showed that waist circumference = 107 cm, hip circumference = 102 cm, and waist-hip ratio = 1.05 (central obesity).

**Figure 1:**
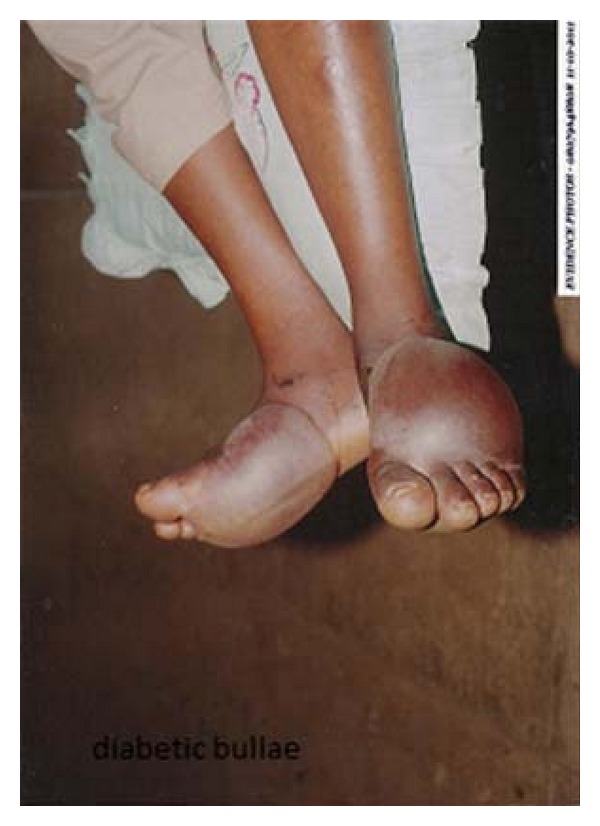


Dorsal and plantar surface of left the foot contained 10 cm × 12 cm bullae each, and the right foot had a 14 cm × 14 cm bullae on the plantar aspect extending to the anteromedial aspect all containing clear fluid, nontender, no differential warmth, and no redness (Figure 1). Dorsalis pedis pulses were full bilaterally, and the pattern of sensory loss was of “gloves and stocking” distribution.

Pulse rate is 88 bpm, blood pressure is 140/90 mmHg, apex beat is at the 5th left intercostals space 2 cm lateral to the midclavicular line, with left ventricular heave, heart sounds were normal S1 and loud A2.

Blood glucose (fasting = 6.1 mmol/L, 2 hours postprandial = 11.1 mmol/L), urinalysis negative for protein, ketones, glucose, nitrite with normal pH.

Parked cell volume = 27%, white blood cell = 11.0 × 10^9^ with neutrophil 56%, lymphocyte 40% monocyte 4%.

Serum urea electrolytes, with urea = 22 mmol/L, sodium = 135 mmol/L, potassium = 5.2 mmol/L, chloride = 102 mmol/L, bicarbonate = 21 mmol/L.

Serum total cholesterol = 7.1 mmol/L, low density lipoprotein cholesterol = 4.1 mmol/L, high density lipoprotein cholesterol = 0.6 mmol/L, triglycerides = 5.3 mmol/L, TC : HDL ratio = 11.8.

The right foot bullae ruptured spontaneously with purulent fluid exuded on the 5th day post-bullae formation, while the left foot was surgically drained following suppuration. Patient was empirically placed on flucloxacillin. Culture of aspirate revealed Staphylococcus aureus. 

Serial sections of tissue biopsy shows hyperkeratotic focally acanthotic pigmented epidermis with subcorneal separation of the granular layer of the epidermis by aggregates of viable and nonviable polymorphs and lymphocytes. There is mild acantholysis of the epidermis. The dermis is fibrocollagenized and moderately infiltrated by lymphocytes.

## 3. Case 2

O. J. is a 47-year-old lady admitted on account of a two-week history of bilateral foot ulcer which started as a swelling on the dorsum of the foot following a 6-hour journey in a tightlypacked bus. Swelling was painless and spontaneously ruptured one week later with clear fluid discharge which became purulent later. There is history of feeling of numbness in both feet for the previous 6 months and no history of intermittent claudication. Feeling of numbness and then progressive darkening of the right 2nd, 3rd, 4th, and 5th toes develop few hours following the return journey, 2 weeks post-bullae formation. The patient is a known diabetic for 8 years but not regular on medications or clinic visit. She was diagnosed hypertensive in the course of the present illness. Her elder sister is diabetic hypertensive, while her mother is hypertensive. She has one child alive; last child 15 years ago was macrosomic and macerated stillbirth. She has never smoked a cigarette and there is no significant alcohol intake.

**Figure 2:**
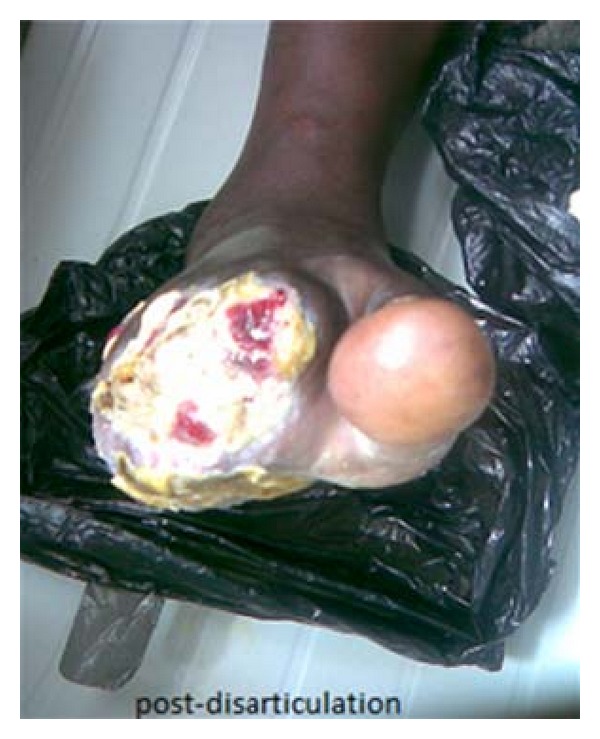


Anthropometry showed that weight = 98 kg, BMI = 35 kgm^−2^ (central obesity) waist-hip ratio = 0.9. Healing ulcer 4 cm × 6 cm on the lateral aspect of the dorsum of both feet, dry gangrene of 2nd, 3rd, 4th and 5th right toes (Figure 2), dorsalis pedis and posterior tibial pulsation full volume on both left and right foot with sensory loss in stocking distribution.

Pulse rate = 96 bpm, regular, full volume; blood pressure = 160/90 mmHg, apex beat not displaced, heart sounds were normal S1 S2.

Spot blood glucose = 16.9 mmol/L; urinalysis showed glucose +++, erythrocyte +++; Serum urea & electrolytes showed, urea = 2.1 mmol/L, sodium = 130 mmol/L, potassium = 4.6 mmol/L, chloride = 92 mmol/L, bicarbonate = 002028 mmol/L.

She was placed on insulin, empirical antibiotic (ceftriazone, and clindamycin), amlodipine, lisinopril, vasoprin.

## 4. Discussion

Bullous disease of diabetes (bullosis diabeticorum) blisters occur spontaneously and abruptly, often overnight, and usually without known antecedent trauma. These patients developed bilateral bullae spontaneously following tightlypacked 10-hour and 6-hour journeys, respectively.

These blisters tend to be asymptomatic, although mild discomfort or burning has been described as experienced by our own cases.

Common findings of bullous disease of diabetes (bullosis diabeticorum) include tense, nontender blisters arising on nonerythematous skin as seen in our patients. The pathophysiology of bullous disease of diabetes (bullosis diabeticorum) is likely multifactorial [1]. Patients with diabetes have been shown to have a lower threshold for suction-induced blister formation compared with nondiabetic controls [5], and because of the acral prominence of diabetic bullae as seen in our patient, the role of trauma has been speculated [1]. Our patient however developed bilateral bullae after long-distance journeys in tightlypacked buses suggesting that prolonged pressure on the proximal lower limbs, vibration, and mild/moderate heat may have played roles. The presence of digital gangrene in the 2nd case without historical or physical evidence of peripheral vascular disease suggests an acute and spontaneous cause likely due to prolonged Raynaud's phenomenon precipitated by vibratory movements in the bus on a background of peripheral neuropathy (involvement of nervi vasorum). The lack of classical pain of Raynaud's phenomenon in these cases can be explained by the presence of neuropathy.

Electron microscopic evidence has also suggested an abnormality in anchoring fibrils. However, this alone does not explain the often spontaneous development of multiple lesions at several locations [1].

In some patients, blisters are related to UV exposure, especially in those with nephropathy [1]. Individuals with end-stage renal disease may have mildly elevated plasma porphyrin levels, possibly contributing to the total pathogenesis of blister formation [1].

 Glycemic control does not appear to have a direct correlation with bullae formation [1]. 

Many, but not all, patients with bullous disease of diabetes have nephropathy or neuropathy [1] as shown in our case. Some authors have hypothesized an etiologic association, possibly related to a local subbasement membrane zone connective-tissue alteration [1]. Hyalinosis of small vessels noted on biopsy specimens has led some authorities to speculate microangiopathy-associated blister induction [1]. In some, especially in patients with neuropathy, UV exposure is also thought to play a role [6].

Bullous disease of diabetes (bullosis diabeticorum) blisters typically heal spontaneously, within 2–6 weeks, as seen in our 2 cases, but lesions often recur in the same or a different location [1]. Although secondary infection may develop as seen in our cases, the prognosis for bullous disease of diabetes is typically good [1].

Although bullous diseases of diabetes lesions often heal without significant scarring, they may be recurrent and also may lead to ulceration [6]. There have also been reports of osteomyelitis arising at a site of bullous disease of diabetes [7] and reports of amputation due to infection [8]. One of the bullae in the 1st case ruptured spontaneously after a week, while the other one got secondarily infected after one week before incision and drainage of the pus was carried out. Healing took place within 4 weeks. The bullae in the 2nd case ruptured spontaneously within a week and healed within 2 weeks, though stayed longer in the hospital due to digital gangrene in the right foot which were disarticulated and subsequently dressed.

Blisters tend to be large (from 0.5–17 cm in diameter), often with an irregular shape, simulating a burn. Some blisters may also be flaccid. 

Although blisters typically occur on the feet or lower legs as described in our cases, rarely, nonacral sites (e.g., trunk) may be involved.

### 4.1. Differential Diagnosis

- Bullous pemphigoid.
- Burns, chemical or electrical.
- Coma blister.
- Drug-induced bullous disorders.
- Epidermolysis bullosa.
- Epidermolysis bullosa acquisita.
- Friction blisters.
- Porphyria cutanea tarda.
- Pseudoporphyria.

Cultures are only warranted if secondary bacterial infections are suspected. In the 1st patient, the fluid in one of the bullae became cloudy within 4 days and the fluid was aspirated using a narrow bore needle and sent for culture. Culture yielded Staphylococcus aureus.

**Immunofluorescence**

No primary immunologic abnormality is noted in bullous disease of diabetes. Although nonspecific capillary-associated immunoglobulin M and C3 have been reported, albeit rarely [9], immunofluorescence findings have not been consistently reproduced by others, and direct immunofluorescence findings are usually negative [10]. However, immunofluorescence studies may be required to exclude clinically similar conditions (e.g., bullous pemphigoid, epidermolysis bullosa acquisita, and porphyrias) that typically show deposition of C3 and immunoglobulin G along the basement membrane zone [1]. Shave biopsy or excisional/incisional biopsy can help to help distinguish bullous disease of diabetes from clinically similar conditions.

For routine histologic sections, the clinician should include the blister and portions of the underlying dermis in the biopsy specimen and submit it in formalin as done in this patient.

Histologic features of bullous disease of diabetes are not entirely specific; lesions have a heterogeneous histologic presentation. 

Serial sections of tissue biopsy from our first case show hyperkeratotic focally acanthotic pigmented epidermis with subcorneal separation of the granular layer of the epidermis by aggregates of viable and nonviable polymorphs and lymphocytes. There is mild acantholysis of the epidermis. The dermis is fibrocollagenized and moderately infiltrated by lymphocytes.

Many of the reported cases describe a separation in the superficial epidermis within the superficial part of the spinous layer [1].

**Figure 3:**
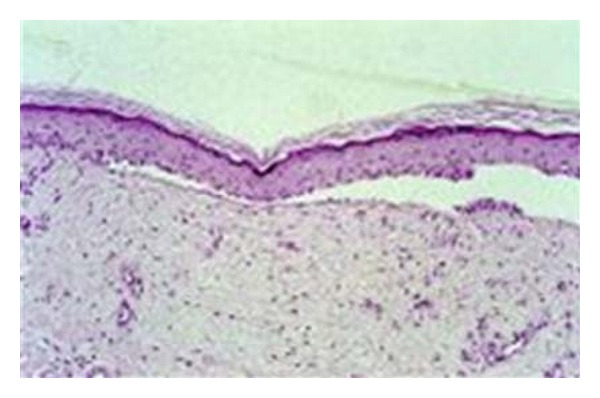
Histology of bullosis diabeticorum showing a noninflammatory blister with a subepidermal and focally intraepidermal separation (hematoxylin and eosin stain) [1].

The blister plane may also appear in a subcorneal, intraepidermal, or subepidermal location; electron microscopy of fresh blisters has revealed separation in a subepidermal location, residing in the lamina lucida or the sublamina densa [11].Anchoring fibrils and hemidesmosomes are reported absent or decreased in early blisters (see Figure 3).

Note that the variable blister plane may be related to the blister age, because reepithelialization can occur within days of blister onset. The blister cavity contains sterile proteinaceous fluid; an inflammatory component is absent or insignificant.

**Figure 4:**
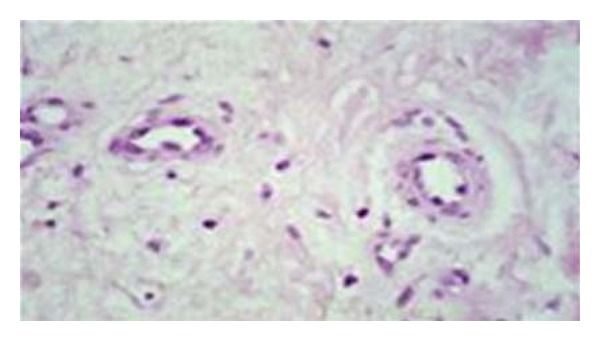
High-power view of the dermis beneath the blister showing capillary wall thickening (hematoxylin and eosin stain) [1].

Surrounding epidermis does not show significant change however, rare reports describe associated spongiosis and degenerative keratinocytic pallor [1]. Acantholysis is absent. Dermal changes (e.g., capillary wall thickening and dermal sclerosis) may reflect the patient's underlying diabetes mellitus (Figure 4) [1]. Caterpillar bodies typical of porphyria have been reported in lesions of bullous disease of diabetes. 

### 4.2. Treatment

Specific treatment of bullous disease of diabetes (bullosis diabeticorum) is unnecessary because the condition is self-limiting [1]. The blister should be left intact whenever possible to serve as a sterile dressing and to avoid secondary infection.

Drug therapy (i.e., antibiotics) is only warranted when secondary staphylococcal infection is present.

Aspiration of fluid from bullous disease of diabetes lesions with sterile technique using a small-bore needle may prevent accidental rupture. Immobilization may prevent damage to the blister. Secondary tissue necrosis may necessitate debridement and possible tissue grafting.

Aggressive wound healing intervention, as enacted with diabetic ulcers, is critical, should the blister become unroofed.

Patients with confirmed bullous disease of diabetes should be monitored for development of secondary infection until lesions heal entirely.

Lipsky and colleagues [12] reported 12 patients with typical diabetic bullae over an 8-year period in Veterans Affairs Medical Center Clinic. The patients were mostly elderly, all but one had lesions located on the lower extremities, all had peripheral neuropathy as seen in our own patients, two had secondary staphylococcal infection of their bullae as seen in one of our cases, and in all patients the lesions healed without scarring. Although most of the patients had had previous similar lesions, the diagnosis of diabetic bullae had not been previously reported in any of them. They concluded after all that diabetic bullae are a not so rare cutaneous disorder.

Bernstein and colleagues [13] reported a patient who had diabetes and experienced twoepisodes of bullae associated with intense, ultraviolet lightexposure. Negative immunofluorescence, early disappearance ofanchoring filaments and half-desmosomes between cell membraneand basal lamina, and the absence of urinary uroporphyrins separatethis entity from certain similar appearing conditions. They concluded cation imbalance, precipitated by renal failure, could be a possiblecausal factor.

## 5. Conclusion

Long journeys by road is a possible predisposing factor to diabetic bullae formation especially on a background of peripheral neuropathy as seen in our cases. The specific role that may be played by pressure, mild to moderate heat, and vibration vis-à-vis Raynaud's phenomenon still remains to be explored. Diabetic patients especially those with peripheral neuropathy should be advised to be cautious while traveling long journeys by road.

## References

[1] Poh-Fitzpatrick MB, Elston DM, Junkins-Hopkins JM (2012). *Bullous Disease of Diabetes*.

[2] Kramer DW (1930). Early or warning signs of impending gangrene in diabetes. *Journal of Medical Record*.

[3] Rocca FF, Pereyra E (1963). Phlyctenar lesions in the feet of diabetic patients. *Diabetes*.

[4] Cantwell AR, Martz W (1967). Idiopathic bullae in diabetics. Bullosis diabeticorum. *Archives of Dermatology*.

[5] Bernstein JE, Levine LE, Medenica MM (1983). Reduced threshold to suction-induced blister formation in insulin-dependent diabetics. *Journal of the American Academy of Dermatology*.

[6] Larsen K, Jensen T, Karlsmark T, Holstein PE (2008). Incidence of bullosis diabeticorum—a controversial cause of chronic foot ulceration. *International Wound Journal*.

[7] Tunuguntla A, Patel KN, Peiris AN, Zakaria WN (2004). Bullosis diabeticorum associated with osteomyelitis. *Tennessee Medicine*.

[8] Lipsky BA, Baker PD, Ahroni JH (2000). Diabetic bullae: 12 cases of a purportedly rare cutaneous disorder. *International Journal of Dermatology*.

[9] James WD, Odom RB, Goette DK (1980). Bullous eruption of diabetes mellitus. A case with positive immunofluorescence microscopy findings. *Archives of Dermatology*.

[10] Basarab T, Munn SE, McGrath JM, Russell-Jones R (1995). Bullosis diabeticorum. A case report and literature review. *Clinical and Experimental Dermatology*.

[11] Toonstra J (1985). Bullosis diabeticorum. Report of a case with a review of the literature. *Journal of the American Academy of Dermatology*.

[12] Lipsky BA, Baker PD, Ahroni JH (2000). Diabetic bullae: 12 cases of a purportedly rare cutaneous disorder. *International Journal of Dermatology*.

[13] Bernstein JE, Medenica M, Soltani K, Griem SF (1979). Bullous eruption of diabetes mellitus. *Archives of Dermatology*.

